# Vascular function in patients with advanced heart failure and continuous-flow or pulsatile ventricular assist devices

**DOI:** 10.1007/s00392-024-02519-x

**Published:** 2024-08-21

**Authors:** Valentina A. Rossi, Delia Nebunu, Matthias P. Nägele, Jens Barthelmes, Thomas Haider, Natallia Laptseva, Konstantinos Bitos, Leonie Kreysing, Michelle Frank, Frank Enseleit, Markus J. Wilhelm, Omer Dzemali, Frank Ruschitzka, Isabella Sudano, Andreas J. Flammer

**Affiliations:** 1 Department of Cardiology, University Hospital Zurich, University of Zurich, Zurich, Switzerland; 2 Clinic for Cardiac Surgery, University Hospital Zurich, University of Zurich, Zurich, Switzerland

**Keywords:** Endothelial function, Ventricular assist device, Retinal vessel analysis, Pulsatile blood flow, Advanced heart failure

## Abstract

**Background:**

A significant proportion of patients with heart failure (HF) progress to an advanced stage, which is associated with a substantial increase in morbidity and mortality. These patients may be eligible for advanced treatment strategies such as mechanical circulatory support with ventricular assist devices (VAD). Vascular dysfunction is a hallmark of heart failure pathophysiology and prognosis. However, whether and to what degree the hemodynamic benefits of VADs influence vascular function remain unknown.

**Methods and results:**

In this study, we evaluated endothelial vascular function with flow-mediated vasodilatation (FMD) and with flicker-light induced retinal vasodilatation (FID). 34 patients with a VAD (age 58 ± 10 years, 85% male, 74% ischemic heart disease, 26 continuous-flow (CF)-LVAD, and 8 pulsatile biventricular (bi)-VAD) were compared to 34 propensity-matched patients (mean age 62 ± 9 years, 68% male, 59% ischemic heart disease) with advanced HF (AdvHF). Endothelial function of larger arteries (FMD) was significantly better in patients after VAD implantation compared to matched AdvHF patients (7.2 ± 4.6% vs. 5.0 ± 3.2%, *p* = 0.03), whereas microvascular arteriolar function (FIDart) did not differ (0.99 ± 1.43% vs. 1.1 ± 1.7%, *p* = 0.78). The arterio-venous ratio (AVR) was higher in the VAD group (0.90 ± 0.06 vs 0.85 ± 0.09, *p* = 0.01), reflecting wider retinal arteriolar and narrower venular diameters. There was no difference in vascular function between patients with CF-LVAD and pulsatile Bi-VAD.

**Conclusion:**

In patients with advanced heart failure, VAD implantation was associated with better endothelial function at the level of large arteries, but not in the microcirculation.

**Supplementary Information:**

The online version contains supplementary material available at 10.1007/s00392-024-02519-x.

## Introduction

Patients with advanced heart failure (HF) have a poor prognosis and present with relevant impairment of quality of life, frequent hospitalizations, and increased mortality rates [1]. Specific therapy for patients with refractory end-stage HF includes intravenous vasodilator and inotropic therapy, mechanical circulatory support, heart transplantation, and palliative care [2]. Among mechanical circulatory support, ventricular assist device (VAD) implantation as a bridge to heart transplantation or as destination therapy represents the best treatment option for long-term survival [3].

The role of endothelial dysfunction in the pathophysiology of HF has increasingly been acknowledged, and a continuum of vascular damage across the whole spectrum of cardiovascular disease, i.e., from cardiovascular risk factors to overt coronary artery disease up to heart failure, has been demonstrated [4]. Importantly, the degree of endothelial impairment has been related to adverse outcomes in advanced HF patients. [5].

Vascular function can be measured non-invasively by flow-mediated dilation (FMD), which represents one of the best-established methods to investigate nitric-oxide (NO)-dependent endothelial function at the level of conduit arteries [6–8], and by means of retinal vessel analysis (RVA), which represents a surrogate for vascular function of smaller vessels where vasodilatory response is modulated, although not entirely mediated, by NO-related pathways [9, 10].

VADs represent an established therapy for advanced HF and allow a sufficient cardiac output restoration. However, their implantation is associated with a high rate of cardiovascular and inflammatory consequences [11]. Continuous-flow left-ventricular VAD (CF-LVAD) represent the most commonly implanted devices and have been associated with a lower rate of complications as compared to the pulsatile ones [12]. Of note, patients implanted with pulsatile biventricular VAD have generally a more compromised hemodynamic state as compared to patients treated with CF-LVAD.

Until now, only a few studies have examined the effects of VAD implantation on endothelial function with controversial results [13]. The aim of this study was, therefore, to investigate vascular function in VAD patients compared to similar patients with advanced heart failure without mechanical support. Further, the impact of the pulsatility of the VAD on endothelial function was assessed.

## Methods

### Study protocol

**Fig. 1:**
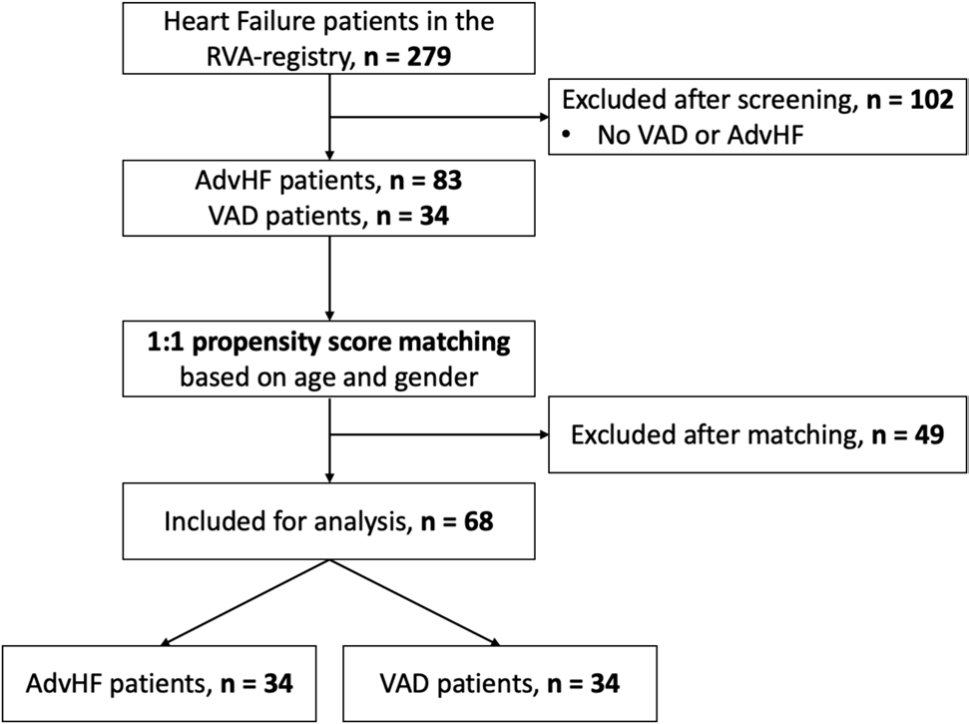
Screening flowchart. AdvHF, advanced heart failure; HF, heart failure; VAD, ventricular assist device

In this cross-sectional, observational study, 34 patients with VAD as bridge to transplantation or destination therapy were included at the University Hospital of Zurich between 2013 and 2021. 34 patients with advanced heart failure (AdvHF) were selected as control group. A propensity score matching based on age and gender was run (1:1 matching, a priori value for calipers: 0.05, R Software, R Studio V1.4.1103, RStudio, PBC) to select patients in the control group among those who presented with following criteria: presence of symptoms (NYHA class III or NYHA IV) despite optimal medical treatment, or symptoms NYHA class II and ≥ 2 hospitalizations due to decompensated HF within the last 12 months together with one of the following: evidence of severely impaired left-ventricular function defined as left-ventricular ejection fraction (LVEF) ≤ 30% or severe left-ventricular (LV) diastolic dysfunction including systolic pulmonary arterial pressure > 35 mmHg, or evidence of reduced exercise capacity of cardiac origin [1]. A patient screening flowchart is presented in Fig. 1.

All participants provided written informed consent prior to enrollment and were instructed to fasten for at least 8 h before the visit (except water), take their regular medication as planned (except for antidiabetic medications), refrain from coffee, alcohol and cigarette consumption for at least 12 h, avoid unusual exercise the day before the examination, and only present in stable medical state (i.e., free of infections or acute illnesses). The study visit was always conducted in the morning to avoid differences in circadian rhythms.

The study protocol was approved by the local ethics committee (KEK-ZH-No. 2014–0329).

### Assessment of macrovascular endothelial function: flow-mediated dilation (FMD)

Flow-mediated dilatation (FMD) was measured with high-resolution ultrasound and defined as peak percent dilatation from brachial conduit artery baseline diameter using established protocols [7]. To ascertain the degree of endothelial-independent vasomotion in both the VAD group and advHF control group, the dilatation of the artery in response to a dose of glycerol trinitrate 0.4 mg (GTN) was measured, as well. The HF cohort performed the latter after excluding relevant hypotension (systolic blood pressure, SBP < 100 mmHg while lying) with an automatic blood pressure device. GTN was not performed in VAD patients due to the unreliability of systemic blood pressure measurements by automated blood pressure devices before and after the administration of nitrates. The reproducibility of our laboratory measurements has been previously published [14].

### Assessment of microvascular endothelial function: retinal vessel analysis (RVA)

Retinal vessel analysis (RVA) was carried out in a standardized manner according to previous published protocols [4, 15]. Briefly, an Imedos Dynamic Retinal Vessel Analyzer (Imedos, Jena, Germany) with a Zeiss FF450 camera (Carl Zeiss Meditec AG, Jena, Germany) was utilized. One eye was randomly selected, and mydriasis was induced using 0.5% tropicamide. Dynamic RVA was conducted to measure retinal arteriolar and venular dilation after provocation with a 12.5 Hz optoelectronic flicker light [16]. Analysis was performed on temporal segments of retinal arteriole and venule irrigating the macula, between 0.5 and 2 optic disc diameters away from the optic disc. The protocol consisted of a 50 s baseline and three 20 s flicker stimulations each followed by a recovery period of 80 s. After the acquisition, the results from the three flicker periods were averaged and the percent dilatation of arteriole or venule from baseline (FIDart and FIDven, respectively) was calculated automatically using the Imedos analysis software. For static RVA, 50 monochromatic fundus photographs were obtained using Visualis and VesselMap2 software (Imedos, Jena, Germany) and retinal arteries and vein diameters in the area 0.5–1 optic disc diameters distant from the optic disc were added with calculation of the central retinal artery and vein equivalent (CRAE and CRVE) [17]. Both values were used to calculate the arterio-venous ratio (AVR = CRAE/CRVE).

### Laboratory assessment

Blood samples were obtained in the fasted state using heparin plasma vials at the beginning of the study visit and analyzed on the same day at the Institute of Clinical Chemistry, University Hospital Zurich using the standard institutional procedures. Undetectable values were replaced by half the lower limit of detection.

### Echocardiography

Transthoracic echocardiography was obtained during regular clinical outpatient visits independent of the study within 12 months from vascular function assessment.

### Follow-up

A follow-up visit was performed in 8 of 34 (23.5%) VAD patients enrolled in the study.

### Statistical analysis

Data were analyzed by R Software (R Studio V1.4.1103, RStudio, PBC) and IBM SPSS Statistics (V.26.0) statistical packages and were collected using REDCap electronic data capture tools (V.12.4.12, 2022, Vanderbilt University) hosted by Clinical Trials Center Zürich [18]. Normality of the data was assessed visually with Q-Q plots and histograms. Continuous data with normal distributions are presented as mean ± standard deviation (SD) and non-normally distributed continuous variables are presented as median and interquartile range (IQR). Categorical data are represented as frequencies and percentages. An unpaired independent t test or independent-samples Kruskal–Wallis test was used to compare vascular parameters between AdvHF and VAD groups. Fisher’s exact test was used to compare the categorical variables between groups. Differences were considered significant when p < 0.05.

## Results

### Baseline characteristics

Baseline characteristics are presented in Table 1. Among the 34 VAD patients, *n* = 26 were CF-LVAD (*n* = 5 HeartMate 3, Abbott, Abbott Park, IL, USA, and *n* = 21 HeartWare HVAD, Medtronic Inc., Framingham, MA, USA), and n = 8 were pulsatile flow-biventricular VAD (Bi-VAD: Berlin Heart EXCOR, Berlin Heart GmbH, Berlin, Germany). No significant differences in gender, age, mean arterial pressure, heart rate, or body mass index nor in the etiology of cardiomyopathy were found (Table 1). VAD patients were more on anticoagulation or aspirin therapy than the Adv-HF group but were less likely to be under betablocker and mineral-receptor antagonists. No differences regarding other renin-angiotensin-blocking therapies, loop diuretics, thiazides, or calcium-antagonists were found. High-sensitive troponin-T, C-reactive protein, and thrombocytes were higher, whereas hemoglobin was lower in the VAD as compared to AdvHF patients.

**Table 1:** Baseline characteristics in AdvHF after matching vs. VAD patients

	Advanced HF *N* = 34	VAD *N* = 34 Bi-VAD/CF-LVAD 8/26	P value
Clinical parameters			
Gender—male, N (%)	23 (68%)	29 (85%)	0.086
Age—years	62 (± 9)	58 (± 10)	0.200
NYHA class			0.400
NYHA 2	10 (29%)	13 (38%)	
NYHA 3	24 (71%)	19 (56%)	
BMI (kg/m^2^)	27.6 (± 4.9)	25.6 (± 4.2)	0.084
MAP (mmHg)	86 (± 12)	85 (± 12)	> 0.9
Smoking			0.200
Never	12 (35%)	10 (29%)	
Ex-smoker	16 (47%)	22 (65%)	
Active	6 (18%)	2 (5.9%)	
Arterial hypertension	17 (50%)	14 (41%)	0.500
Coronary artery disease	20 (59%)	25 (74%)	0.200
Dyslipidemia	18 (53%)	18 (53%)	> 0.9
Diabetes	14 (41%)	12 (35%)	0.600
Medications			
VKA	18 (53%)	34 (100%)	< 0.001
ASS	14 (41%)	26 (76%)	0.003
P2Y12-R antagonist	9 (26%)	10 (29%)	0.800
ACE-I/ARB	24 (71%)	21 (62%)	0.400
Betablocker	31 (91%)	17 (50%)	< 0.001
Loop diuretic	31 (91%)	30 (88%)	> 0.9
MRA	27 (79%)	11 (33%)	< 0.001
Thiazide	7 (21%)	2 (5.9%)	0.150
Calcium channel blocker	4 (12%)	10 (29%)	0.072
Proton-pump inhibitor	19 (56%)	33 (97%)	< 0.001
Statin	24 (71%)	20 (59%)	0.300
Metformin	8 (24%)	3 (8.8%)	0.100
Laboratory parameters			
eGFR (mL/min/1.73m^2^)	57 (± 24)	54 (± 24)	0.600
CRP, high-sensitive (mg/L)	6 (± 10)	13 (± 13)	< 0.001
Troponin T, high sensitivity (ng/L)	34 (± 25)	77 (± 81)	0.014
NT-proBNP (ng/L)	4′961 (± 8,367)	2′619 (± 2,116)	0.600
Hemoglobin (g/L)	133 (± 16)	112 (± 22)	< 0.001
Thrombocytes (T/L)	219 (± 61)	260 (± 99)	0.056
Leucocytes (G/L)	7.27 (± 2.05)	7.20 (± 2.40)	0.800

Data are mean ± SD or median (IQR 25–75) or n (%), as appropriated

*ACEi* angiotensin-converting enzyme inhibitor, *ARB* angiotensin receptor blocker, *VKA* vitamin K antagonists, *ASS* aspirin, *Bi-VAD* biventricular assist device, *BMI* body mass index, *CF-LVAD* continuous-flow left-ventricular assist device, *CRP* C-reactive protein, *HR* heart rate, *MAP* mean arterial pressure, *MRA* mineralocorticoid receptor antagonist, *NYHA* New York Heart Association functional classification, *NT-proBNP* N-terminal pro-B-type natriuretic peptide

Baseline characteristics of VAD patients based on the type of implanted VAD (CF-LVAD vs. Bi-VAD) are summarized in Table 2. Both in the CF-LVAD (*n* = 17, 65%) and in the Bi-VAD (*n* = 6, 75%) subgroup, most of the devices were implanted as “bridge-to-transplant”. The average time from device implantation to study visit did not differ significantly. Bi-VAD patients were more compromised hemodynamically, thus justifying the implantation of biventricular support devices. Of note, Bi-VAD patients showed a tendency toward lower rate of aortic valve opening and shorter time to transplantation.

**Table 2:** Baseline characteristics of VAD subgroups

	Bi-VAD	CF-LVAD	P value
	*N* = 8	*N* = 26	
Clinical characteristics			
Gender—*male N (%)*	1 (12%)	4 (15%)	> 0.9
Age (years)	51 ± 14	61 ± 7	0.051
MAP (mmHg)	86 ± 10	84 ± 12	0.500
HR (beats/min)	67 ± 5	67 ± 13	0.800
NYHA *II*	3 (38%)	11 (42%)	0.500
NYHA *III*	5 (63%)	15 (58%)	
Etiology of the cardiomyopathy			
Ischemic heart disease	7 (88%)	19 (73%)	0.400
Number of diseased coronary arteries			0.600
*1*	1 (14%)	5 (28%)	
*2*	1 (14%)	5 (28%)	
*3*	5 (71%)	9 (50%)	
Dilated cardiomyopathy	1 (12%)	9 (35%)	0.077
Device			
Implantation strategy			
*Bridge-to-transplantation*	6 (75%)	17 (65%)	0.611
*Destination therapy*	2 (25%)	9 (35%)	
Time from implantation to study visit (months)	7 ± 4	14 ± 12	0.130
Aortic valve opening—*yes (%)*	3 (38%)	17 (68%)	0.200
Outcome			
HT	6 (75%)2	9 (35%)	0.100
Time from VAD implantation to HT (months)	15 ± 8	26 ± 15	0.066
Deaths	3 (37.5%)	8 (30.7%)	0.722

Data are mean ± SD or median (IQR 25–75) or *n* (%), as appropriated

*MAP* Mean arterial pressure, *HR* heart rate, *Bi-VAD* biventricular pulsatile VAD, *CF-LVAD* continuous-flow left-ventricular assist device, *HT* heart transplantation, *VAD* ventricular assist device

### Vascular function in VAD patients compared to patients with advanced heart failure

**Fig. 2:**
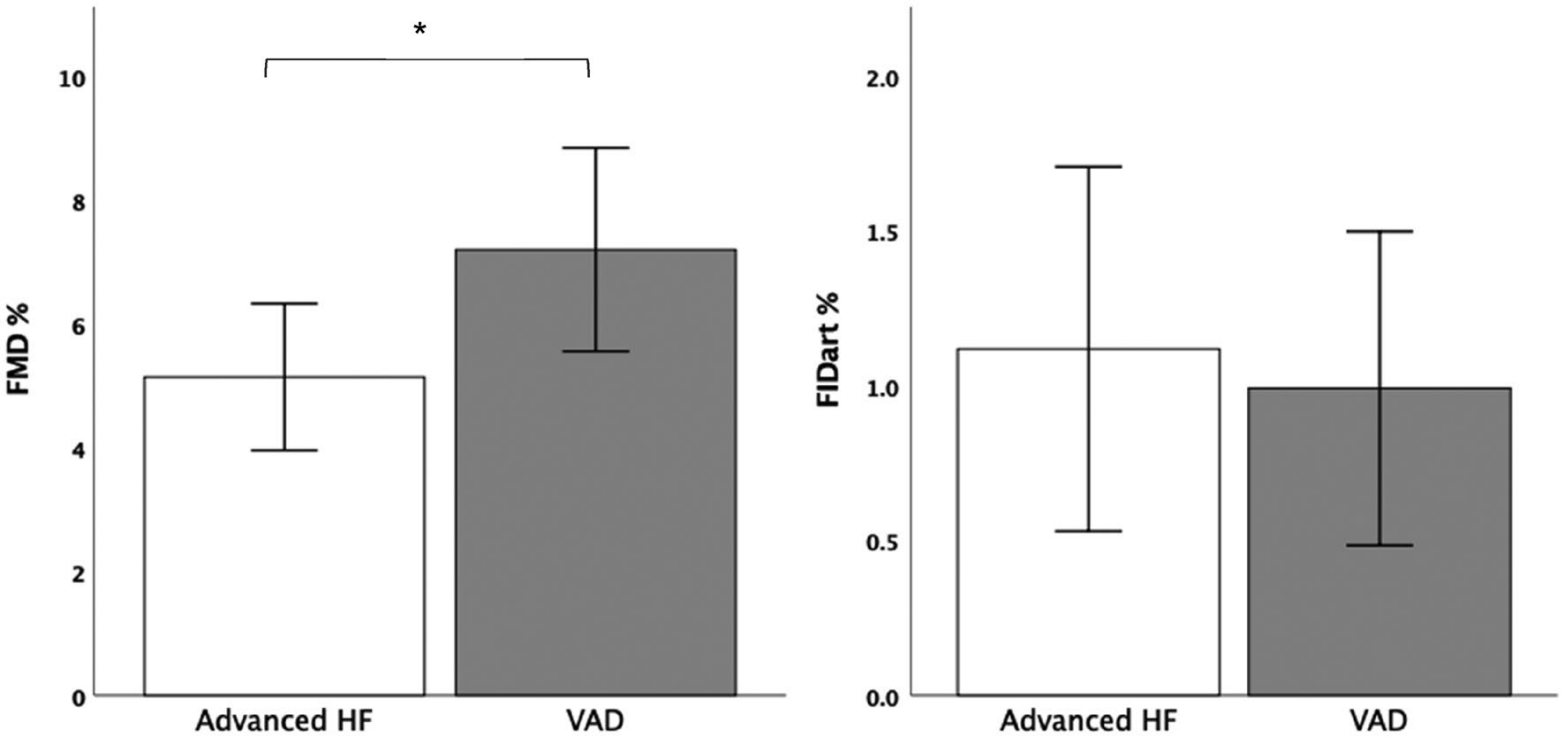
Flow-mediated dilation (FMD) in advanced HF vs VAD. Flow-mediated dilation (FMD) was significantly better in the VAD population (CF-LVAD and Bi-VAD analyzed together). No differences were observed in flicker-light-mediated arteriolar vasodilation (FIDart%). Values are mean (95% confidence interval). *p value < 0.05. FMD, flow-mediated dilation; FIDart, flicker-light-induced retinal arteriolar dilation; HF, heart failure

**Fig. 3:**
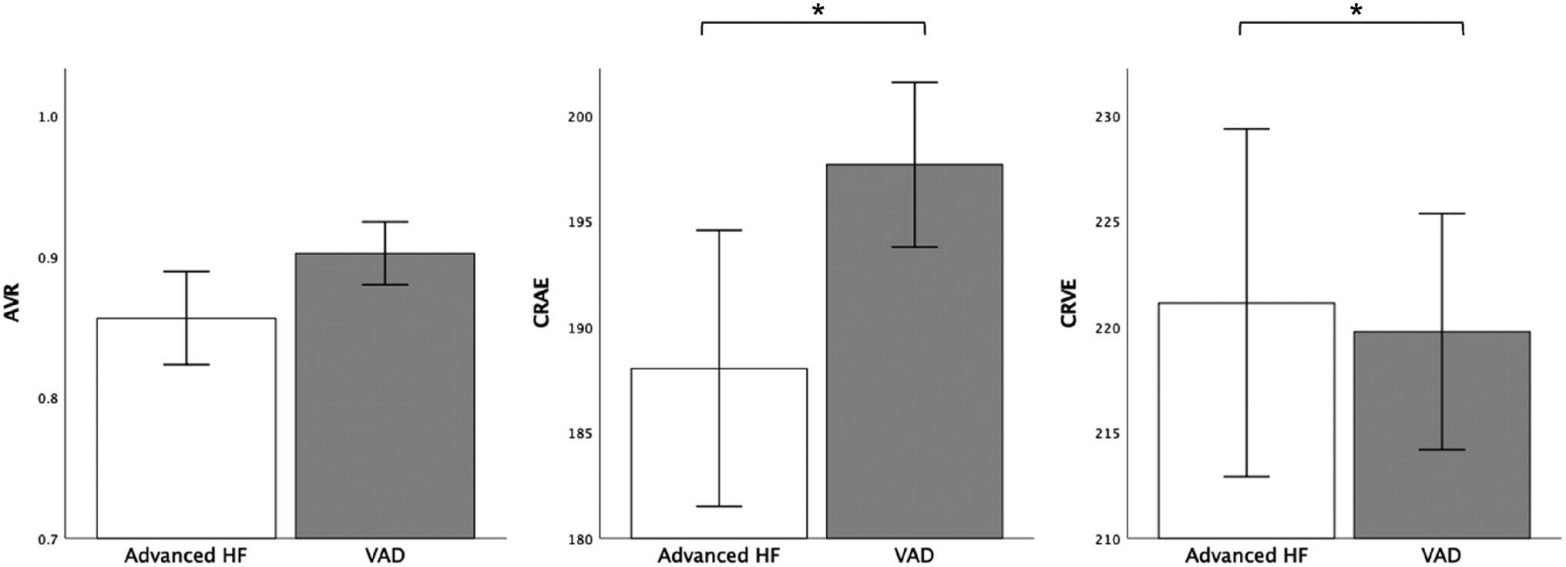
Retinal vessel analysis in patients with advanced HF vs patients with VAD. Comparisons for patients with advanced HF (Adv-HF) vs VAD for arterio-venous ratio (AVR), central retinal arterial equivalent (CRAE), and central retinal venous equivalent (CRVE). Values are mean (95% confidence interval). *p value < 0.05. FMD, flow-mediated dilation; FIDart, flicker-light-induced retinal arteriolar dilation; HF, heart failure

VAD patients had a significantly better endothelial function as assessed with FMD than comparable, matched patients with advanced heart failure without an assist device (FMD 7.2 ± 4.6% and 5.0 ± 3.2%, respectively, *p* = 0.03—Fig. 2, Table 3*)*. Concerning retinal microvascular function, flicker-light induced retinal microvascular vasodilation (FIDart and FIDven) did not differ between the two groups (Table 3, Fig. 2). The ratio between the retinal arterioles and venule diameters (AVR) was significantly increased in patients with VADs as compared to controls (AVR 0.90 ± 0.06 in VAD population vs 0.85 ± 0.09 in AdvHF, *p* = 0.02), because of wider retinal arteries (CRAE 198 ± 11 as compared to 188 ± 18, respectively, *p* = 0.01, Table 3, Fig. 3). Other vascular parameters did not differ between the groups.

**Table 3:** Parameters of endothelial function

	Advanced HF *N* = 34	VAD *N* = 34	P value
FMD			
FMD (%)	5.0 ± 3.2	7.2 ± 4.6	0.030
GTN (%)	15.7 ± 6.1	21.2 ± 7.5	0.064
DVA*—dynamic analysis*			
FIDart (%)	1.10 ± 1.70	0.99 ± 1.43	0.780
FIDven (%)	3.17 ± 1.97	3.29 ± 2.20	0.800
DVA—*static analysis*			
AVR	0.85 ± 0.09	0.90 ± 0.06	0.020
CRAE	188 ± 18	198 ± 11	0.010
CRVE	222 ± 22	220 ± 16	0.630

Data represented as mean ± SD

*AVR* arterio-venous ratio, *CRAE* central retinal artery equivalent, *CRVE* central retinal venular equivalent, *FIDart* flicker-light-induced arterial dilation, *FIDven* flicker-light-induced venous dilation

### Vascular function between patients with continuous-flow and pulsatile-flow VADs

We found no differences in vascular parameters within pulsatile and continuous-flow pumps (Supplementary data, Table 1).

### Follow-up

Among the patients with VADs, we have follow-up data of 8 patients (23.5%) over a median period of 3.1 years [2–4.5]. No differences in endothelial function was found in this small subgroup. Follow-up data are displayed in Supplementary data, Table 2.

## Discussion

This is the first study comprehensively assessing vascular function in various vascular beds in patients with VADs and advanced heart failure. In this study, we found a better endothelial function in large conduit arteries in patients with VADs, whereas vascular function in small-caliber arterioles was not different between the two groups. Despite restoring of a more physiological pulsatile blood flow with Bi-VAD, vascular function did not differ from patients receiving a continuous-flow device.

### Endothelial function in VAD patients versus in patients with advanced heart failure

This is the first study reporting a better endothelial function in large conduit arteries as assessed by FMD in VAD compared to matched patients with advanced HF.

Although this study does not allow delineating exact mechanisms, we hypothesize that the observed FMD improvement in VAD patients is likely due to the enhanced cardio-circulatory status, which improves advanced heart failure. This may lead to a more balanced systemic stress response, thereby potentially increasing the net bioavailability of nitric oxide (NO) [7].

Contrary to our findings, Witman et al. reported impaired FMD in patients on LVAD compared to HFrEF patients with NYHA II and healthy controls [13]. Of note, they included mainly LVAD patients implanted with a second-generation CF-LVAD (HeartMate II) exhibiting axial flow, whereas in our study, most LVAD patients had a newer generation left-ventricular devices, namely HeartMate 3 and HeartWare HVAD, which produces centrifugal flow. Furthermore, they compared VAD patients with well-controlled, stable HFrEF patients with NYHA II, not with patients with advanced heart failure such as in our study. Accordingly, in an analysis comparing VAD patients to patients with advanced HF and NYHA III/IV, no differences in endothelial function were found [13]. Similarly, Cortese et al. found no differences in endothelial function as assessed by FMD in patients implanted with CF-LVAD as compared to advanced HF patients listed for heart transplantation, and C-reactive protein was the only factor which negatively influenced endothelial function [19].

### Effects of VAD pulsatility on endothelial function in large conduit arteries

In our study, the finding of a better endothelial function as measured by FMD in large conduit arteries in VAD patients was irrespective from the presence of VAD-induced blood-flow pulsatility.

The role of pulsatility on endothelial function is debated, as blood flow-generated shear stress is a potent mechanical inductor of nitric-oxide (NO) both in vitro and in vivo [20, 21]. Accordingly, the chronic lack of pulsatility has been associated with increased vascular stiffening in a biochemical model of shear stress induced endothelial NO synthase (eNOS) [20]. Similar findings have been reproduced in animal models, where the absence of blood-flow pulsatility has been related to augmented peripheral vascular resistances despite no changes in serum catecholamines and angiotensin-II concentrations [21]. These in vivo findings suggest that a laminar flow may lead to a better preservation of NO reserve in VAD patients. [21]

In humans, comparisons about the effect of VAD-related pulsatility on endothelial function are affected by the heterogeneity of VAD devices [13, 19, 22]. First-generation VADs were relatively large, paracorporeal pumps generating a pulsatile flow. Second-generation VADs, such as the HeartMate 2, were smaller, intrathoracic devices, which produced a laminar axial flow [23]. Lately, third-generation VADs devices such as the HeartMate 3 and the HeartWare have been launched. The new-generation HeartMate 3 VAD has a fully magnetically levitated rotor and wider blood-flow paths with a centrifugal effect on vessels walls, the HeartWare HVAD is based on a technology with a centrifugal continuous flow producing a pulsatile waveform by interacting with the patient’s patients’ dynamic and physiologic conditions [24]. Each one of these later continuous-flow devices might exert different forces and, thus, differently stimulate vascular walls. Of note, patients who received these devices might have a residual pulsatility because of the contractile reserve of their native ventricles.

In a previous study investigating vascular endothelial function in large conduit arteries via FMD, patients who were implanted with a first-generation, pulsatile LVAD had a better peripheral vascular reactivity as compared to patients who were implanted with second-generation, non-pulsatile CF-VAD devices [22]. These findings support the prioritization of intrinsic pulsatility in patients implanted with axial flow devices. Accordingly, the presence of a pulsatile systemic blood flow has been shown to be advantageous over non-pulsatile blood flow on peripheral circulation, organ function, and metabolism in patients implanted with VAD [25, 26]. Of note, the type of LVAD device has been observed to significantly affect the state of endothelial function more than other clinical factors [26].

In our study, patients treated with CF-LVAD were implanted with newer generation left-ventricular devices with a minimal intrinsic pulsatility, namely HeartMate 3 and HeartWare HVAD. This may explain why we detected a better FMD in VAD patients as compared to patients with advanced HF. Moreover, in our population, a high proportion of patients on CF-LVAD presented on echocardiography with an opening aortic valve (68% of the CF-LVAD), thus increasing the peripheral intrinsic pulsatility despite the presence of continuous-flow devices. This could furthermore explain the lack of differences between CF-LVAD and pulsatile Bi-VAD in our cohort. Furthermore, patients implanted with Bi-VAD usually present with a more compromised hemodynamic function of both ventricles and right-ventricular device support is associated with a higher mortality [27].

### Effects of VAD pulsatility on endothelial function in small-caliber vessels

Flicker-light induced retinal arteriolar dilatation (FIDart) measured by dynamic retinal vessel analysis is a novel marker for assessing microvascular retinal function and has been shown to be affected in patients with heart failure [15]. Indeed, FIDart values in VAD recipients were in line with the previously reported values in HF patients [15] and did not differ compared to the control group. The reason for this discrepancy between better FMD but similarly impaired FIDart as compared to patients with advanced HF is possibly related to the fact that different pulsatility patterns do not influence flicker-light-induced retinal arteriolar vasodilation. Indeed, retinal vessels are anatomically part of the cerebral circulation, and their regulation differs from the arterioles located in other body districts [28]. Indeed, autoregulatory cerebral blood-flow processes are maintained regardless of non-pulsatile cerebral perfusion in patients with VAD implantation. [29]

Interestingly, we found a higher arterio-venous ratio likely due to bigger retinal arteriolar diameters in the VAD group compared to patients with advanced HF despite no differences in the retinal venular diameter. This finding is of interest, since a wider retinal arteriolar diameter has been mainly observed in patients with diabetes, current cigarette smoking and higher plasma fibrinogen levels [30]. VAD patients have a higher baseline prothrombotic status related to the chronic presence of foreign devices and are accordingly therapeutically anticoagulated with vitamin K antagonists. Although the investigated VAD patients were hemodynamically stable during the analyses, they had lower hemoglobin and higher C-reactive protein values. These findings may be explained in the setting of an otherwise subclinical prothrombotic status despite a higher proportion of anticoagulation with warfarin and antithrombotic therapies in VADs. Of note, prothrombotic status in VADs patients is mainly related to protein S and protein C function and activation, which are influenced by proinflammatory states but not warfarin [31]. Similarly, endothelial function was better in VAD patients, although they were less often on guideline-directed heart failure therapy (less betablockers and mineral-receptor antagonists) as compared to the advanced HF cohort. As such, higher C-reactive protein values in VAD patients might not be indicative of the general inflammation state.

## Limitations

This is a single-center, cross-sectional observational study. There was no intra-individual comparison before VAD implantation and our results provided only a snapshot in patient’s individual circulatory state. Residual confounding cannot be excluded. Longitudinal, multicentric studies including a larger number of patients is recommended to confirm our findings. In the investigated population, VAD groups were heterogeneous and pulsatile VAD were only 24% of the population, thus underrepresented to draw confirmative conclusions. Furthermore, the ongoing evolution of VADs makes it difficult to compare our results with the previous studies and it is also possible that the next generation of VAD will provide better hemodynamic conditions with further improvement in endothelial function. Moreover, this study was not designed to investigate the exact mechanisms of the effects of VAD therapy on endothelial function. The small study group and the heterogeneity in the VAD group do not allow for confirmatory mechanistic conclusions.

## Conclusions

This is the first study to demonstrate a remarkably improved endothelial function in conduit arteries in patients with VADs compared to patients with advanced heart failure. However, the vascular function of small-caliber vessels was not different between these two groups. Pulsatile VADs did not show better vascular function than non-pulsatile devices. Further research on this field is warranted, given the constant innovation experienced in the field of mechanical circulatory support.

## Supplementary Information

Below is the link to the electronic supplementary material.

Supplementary file1 (DOCX 19 KB)

## Data Availability

The data that support the findings of this study are available from the corresponding author upon reasonable request.

## Abbreviations

AdvHF: Advanced heart failure
AVR: Arterio-venous ratio
VAD: Ventricular assist device
Bi-VAD: Pulsatile flow-biventricular assist device
CF-VAD: Continuous-flow left-ventricular assist device
CRAE: Central retinal artery equivalent
CRVE: Central retinal vein equivalent
HF: Heart failure
FIDart: Flicker-light mediated retinal arteriolar dilation
FIDven: Flicker-light mediated retinal venular dilation
FMD: Flow-mediated dilation
VAD: Ventricular assist device
