# Supplementary material for: Vascular function in patients with advanced heart failure and continuous-flow or pulsatile ventricular assist devices
Source: Clin Res Cardiol. 2024 Aug 21;114(10):1290–9. doi: 10.1007/s00392-024-02519-x (PMC12460578; doi:10.1007/s00392-024-02519-x)
Supplement: Supplementary file 1 — Supplementary file1 (DOCX 19 KB) [file 392_2024_2519_MOESM1_ESM.docx]

**Supplementary Table 1. Vascular parameters in VAD subgroups**

|  | Bi-VAD  N=8 | CF-LVAD  N=26 | p-value |
| --- | --- | --- | --- |
| Retinal vessel analysis |  |  |  |
| FIDart (%) | 0.95 ±1.03 | 1.00 ±1.55 | 0.900 |
| FIDven (%) | 3.99 ±2.83 | 3.07 ±1.98 | 0.500 |
| AVR | 0.89 ±0.04 | 0.91 ±0.07 | 0.100 |
| CRAE | 196 ±10 | 198 ±11 | 0.700 |
| CRVE | 221 ±13 | 219 ±17 | 0.700 |
| Flow mediated dilation |  |  |  |
| FMD (%)  GTN (%) | 7.7 ±4.1  24±3.9 | 7.1 ±4.8  19±9.3 | 0.700  0.321 |
|  | **HeartMate 3**  N=5 | **HeartWare**  N=21 | **p-value** |
| Retinal vessel analysis |  |  |  |
| FIDart (%) | 1.08 ±1.51 | 0.98 ±1.60 | 0.700 |
| FIDven (%) | 4.28 ±3.01 | 2.77 ±1.60 | 0.400 |
| AVR | 0.89 ±0.11 | 0.91 ±0.06 | 0.500 |
| CRAE | 196 ±18 | 199 ±10 | 0.500 |
| CRVE | 222 ±21 | 219 ±16 | 0.600 |
| Flow mediated dilatation |  |  |  |
| FMD (%) | 6.1 ±3.5 | 7.3 ±5.1 | 0.700 |
| Intermittent aortic valve opening | **Yes**  N=17 | **No**  N=8 | **p-value** |
| Retinal vessel analysis |  |  |  |
| FIDart (%) | 1.18 ±1.75 | 0.71±1.07 | 0.442 |
| FIDven (%) | 2.92 ±1.54 | 3.81 ±2.75 | 0.442 |
| AVR | 0.91 ±0.07 | 0.9 ±0.07 | 0.831 |
| CRAE | 198 ±13 | 198 ±7 | 0.928 |
| CRVE | 219 ±18 | 220 ± 15 | 0.878 |
| Flow mediated dilatation |  |  |  |
| FMD (%)  GTN (%) | 7.5 ±4.1  18.5 ± 7.8 | 6.5 ± 6.6  27.3 ± 3.8 | 0.694  0.079 |

Data represented as mean±SD. GTN% in HeartMate 3 patients was not available, since MAP was too low.

*Abbreviations*: AVR, arterio-venous ratio; CRAE, central retinal artery equivalent; CRVE, central retinal venular equivalent; FIDart, flicker-light induced arterial dilation; FIDven, flicker-light induced venous dilation.

**Supplementary Table 2. Vascular parameters in VAD patients with a follow-up examination**

|  | Baseline data  N=8 | Follow-up  N=8 | p-value |
| --- | --- | --- | --- |
| Retinal vessel analysis |  |  |  |
| FIDart (%) | 1.5 [0.3-5.9] | 1.3 [0.1-6.6] | 0.828 |
| FIDven (%) | 2.6 [2-6] | 3 [1.7-10.3] | 0.483 |
| AVR | 0.75 [0.93-0.88] | 0.88 [0.85-0.95] | 0.154 |
| CRAE | 175 [171-208] | 203 [192-213] | 0.193 |
| CRVE | 223 [206-263] | 226 [214-278] | 0.761 |
| Flow mediated dilation |  |  |  |
| FMD (%)  GTN (%) | 6.2 [3.8-11.1]  10.2 [10.1-27.2] | 8.7 [6.9-15.2]  24.8 [22.5-26.9] | 0.264  0.086 |

Data represented as median±IQR.

*Abbreviations*: AVR, arterio-venous ratio; CRAE, central retinal artery equivalent; CRVE, central retinal venular equivalent; FIDart, flicker-light induced arterial dilation; FIDven, flicker-light induced venous dilation.
